# Snooker Structure-Based Pharmacophore Model Explains Differences in Agonist and Blocker Binding to Bitter Receptor hTAS2R39

**DOI:** 10.1371/journal.pone.0118200

**Published:** 2015-03-02

**Authors:** Wibke S. U. Roland, Marijn P. A. Sanders, Leo van Buren, Robin J. Gouka, Harry Gruppen, Jean-Paul Vincken, Tina Ritschel

**Affiliations:** 1 Laboratory of Food Chemistry, Wageningen University, 6708 WG Wageningen, The Netherlands; 2 Computational Discovery and Design Group (CDD), Centre for Molecular and Biomolecular Informatics (CMBI), Radboud university medical center, 6500 HB Nijmegen, The Netherlands; 3 Unilever R&D, 3133 AT Vlaardingen, The Netherlands; University of Minnesota, UNITED STATES

## Abstract

The human bitter taste receptor hTAS2R39 can be activated by many dietary (iso)flavonoids. Furthermore, hTAS2R39 activity can be blocked by 6-methoxyflavanones, 4’-fluoro-6-methoxyflavanone in particular. A structure-based pharmacophore model of the hTAS2R39 binding pocket was built using Snooker software, which has been used successfully before for drug design of GPCRs of the rhodopsin subfamily. For the validation of the model, two sets of compounds, both of which contained actives and inactives, were used: (i) an (iso)flavonoid-dedicated set, and (ii) a more generic, structurally diverse set. Agonists were characterized by their linear binding geometry and the fact that they bound deeply in the hTAS2R39 pocket, mapping the hydrogen donor feature based on T5.45 and N3.36, analogues of which have been proposed to play a key role in activation of GPCRs. Blockers lack hydrogen-bond donors enabling contact to the receptor. Furthermore, they had a crooked geometry, which could sterically hinder movement of the TM domains upon receptor activation. Our results reveal characteristics of hTAS2R39 agonist and bitter blocker binding, which might facilitate the development of blockers suitable to counter the bitterness of dietary hTAS2R39 agonists in food applications.

## Introduction

Bitter taste is perceived via bitter taste receptors located in taste buds on the tongue. Amongst the 25 human bitter taste receptors (hTAS2Rs), ligands have been identified for 21 hTAS2Rs.[1,2] The bitter taste receptor hTAS2R39 has been identified as one of the sensors of dietary phenolics, comprising the classes of flavonoids and isoflavonoids.[3,4] Many phenolics have been associated with the healthiness of fruits and vegetables, but inevitably also with bitterness, which can affect consumer acceptance of such products. In order to counter this off-taste, different strategies can be employed. Traditionally, undesired bitter taste can be masked by addition of flavors or tastants. A second approach in reducing bitterness is to prevent contact of the bitter compounds with the bitter taste receptor by techniques such as encapsulation, molecular inclusion or complexation. It has been shown that phenolics can be bound to proteins like casein, leading to decreased activation of bitter taste receptor hTAS2R39 and to decreased bitterness perception *in vivo*.[5] A third strategy is the application of so-called bitter blockers (or antagonists). For hTAS2R39, it has been found that 6-methoxyflavanones (the synthetic 4’-fluoro-6-methoxyflavanone, in particular) can decrease the response towards diverse bitter compounds (or agonists).[6] Agonists and blockers can bind to the receptor, but only the binding of agonists results in receptor activation. An intriguing question is whether blockers and agonists have different binding modes to hTAS2R39, provoking different responses in signal transduction. So far, this question has remained unanswered.

Bitter taste receptors belong to the family of G-protein coupled receptors (GPCRs).[7] Amongst the GPCRs, bitter receptors form their own subfamily, denoted ‘taste 2’, and until now, no crystal structure of any bitter taste receptor is available. In recent years, approaches to elucidate the structures of several members of the hTAS2R family by homology modeling have been described, often in combination with molecular docking studies. These included hTAS2R1 [8,9], hTAS2R10 [10], hTAS2R16 [11,12], hTAS2R31 [13], hTAS2R38 [11,14,15,16,17], hTAS2R43 [13], and hTAS2R46 [18,19]. Most publications suggested the presence of a single binding pocket within the trans-membrane (TM) region, consisting of seven TM domains (numbered I to VII), of the respective bitter receptors. Predicted or experimentally determined amino acid residues involved in agonist interaction were reported to be accumulated in TM III, TM VI and TM VII.[20] So far, few bitter taste receptor antagonists have been identified and modeling has scarcely been applied.[6,13,21,22,23,24]

Snooker has been proven to be a powerful structure-based approach to generate pharmacophore hypotheses based on the properties of the receptor. These hypotheses have been used for compounds binding to the extracellular side of the TM domain of the rhodopsin subfamily of GPCRs, and can be applied in principle for all subfamilies of GPCRs.[25] Structure-based modeling has several advantages over ligand-based modeling approaches: i) the model is not biased towards known ligands, ii) a hypothesis of the protein-ligand binding mode can be made, and iii) details about protein-ligand interactions can be retrieved.

Here, we apply Snooker to build a structure-based pharmacophore of hTAS2R39. This pharmacophore model is purely built on the information obtained from many taste receptor amino acid sequences and a GPCR receptor template. Using only the sequence conservation and evolution theory, important amino acids can be identified, and their involvement in ligand-binding or in the receptor activation mechanism can be characterized.[26] Pharmacophore models are derived from this information and do not include compound information. The validation of the obtained pharmacophore model is based on experimental data of previously published compounds. Based on the structure-based pharmacophore, the interaction between the receptor and agonists or blockers can be described. Using this approach, the importance in ligand binding of certain amino acids that are conserved amongst TAS2R39 homologs is demonstrated, and differences in binding modes between blockers and agonists were identified.

## Methods

### Snooker

Snooker was previously developed and applied for rhodopsin-subfamily GPCRs and uses only receptor information to build a pharmacophore model of the protein-ligand binding site. In the current manuscript Snooker is extended to bitter receptors, a different class of GPCRs.[25] The approach is semi-automated and constructs a homology model of the transmembrane domains. Furthermore, it prioritizes amino acids on the probability of being involved in ligand–binding, based on an analysis of conserved residues in a multiple sequence alignment.[26] Subsequently, protein features are converted to ligand space, and pharmacophore features are generated at ‘hot-spots’ of interaction features. In order to use Snooker on bitter receptor hTAS2R39, a multiple sequence alignment of the taste receptor family, including many different species, was obtained from GPCRDB (**S2 File**)[27] and uploaded to Snooker. In total 711 sequences were introduced to Snooker. The definition of the transmembrane helices of the GPCR was manually compared to other publications [10,18,28] and no significant differences were observed. Snooker combines several approaches and consists of eight steps:

1. Template selection: The known GPCR crystal structure of bovine rhodopsin with PDB-code 1GZM was used as a template for modeling.[29] For Snooker, external and internal loops are not considered, resulting in a model of only the transmembrane helices.
2. Homology model construction: The homology model was constructed based on the alignment of the TM domains with the template sequence. Snooker keeps the backbone of the TM as in the template structure. Ballesteros and Weinstein numbering was used for the numbering of the amino acid residues in the TM domains. This nomenclature uses the principle that the helices are numbered from 1 to 7, and that the most conserved amino acid residue in each helix receives the number 50. Amino acid residues are counted downwards in N-terminal direction and upwards in C-terminal direction. Ballesteros and Weinstein numbers of transmembrane helices are: TM I: 1.33–1.56; TM II: 2.40–2.65; TM III: 3.25–3.51; TM IV: 4.43–4.64; TM V: 5.38–5.63; TM VI: 6.37–6.59; TM VII: 7.34–7.56. The alpha-helices of GPCRs are positioned in the membrane which requires hydrophobic amino acid side chains facing towards the membrane. Therefore, the beginning of TM V was shifted by three amino acids to ensure that hydrophobic amino acids were pointing to the membrane and hydrophilic residues were pointing to the inside of the receptor.
3. Rotamer sampling: The rotamers indicate the likelihood of a direction of the amino acid residue side chains, which enables the calculation of the most probable binding pocket. Default Snooker rotamers and rotamer scores were added to the initial homology model to account for possible model inaccuracy of the initial homology model. Use of an ensemble of rotamers avoided the computational magnitude that would result from considering all possible models, which can be obtained by combining all possible rotamer states for each single residue. The rotamer ensemble and likelihood of occurrence of each rotamer was subsequently used to generate both the protein- and pocket volume definition and interaction features.
4. Protein- and pocket volume definition: Because default Snooker parameters for cavity detection resulted in a very small pocket, all tetrahedra with edges longer than 8.0 Å instead of the default value of 8.5 Å were removed. All other parameters were used at default settings.
5. Residue scoring: Residues were scored upon ligand binding probability.[26] To determine the ligand binding residues specific for this subfamily, a subfamily definition of receptors q50kk9_9prim, q50kl0_pantr, q50kl2_human, q5ug17_pantr, t2r39_human, t2r39_macmu, t2r39_panpa, and t2r39_pantr was used. Examples for the use of the multiple sequence alignment tree are shown in **Fig. A in S1 File**. The subfamily definition of receptors is shown within the yellow mark in **Fig. A in S1 File**.
6. Placement of interaction features: Interaction features were positioned for each rotamer inside the pocket volume according to the rotamer likelihood and ligand binding likelihood of the residue. Both have been performed using default settings.
7. Pharmacophore hypothesis generation: Interaction feature densities were clustered to generate pharmacophore features. All features were generated using a fuzzy pharmacophore algorithm with an Rc value of 2.5 Å.[30] The top three features for hydrophobic, donor and acceptor properties were selected and comprise the final pharmacophore.
8. Pharmacophore validation: Three validation sets (see Compound datasets and preparation) were screened with all possible pharmacophore feature combinations containing 5 out of 9 or 6 out of 9 features, to identify the compounds that fit into the pharmacophore model.

### Compound datasets and preparation

Three sets of compounds were used for validating the structure-based pharmacophore model: lab set, literature set, and blockers. The first two sets included hTAS2R39 agonists and compounds which were inactive on hTAS2R39; the last set included so-called hTAS2R39 blockers. The *lab set* (**Table A in S1 File**) is a set of flavonoids (2-phenyl benzopyrans) and isoflavonoids (3-phenyl benzopyrans) tested for activation of bitter receptor hTAS2R39 in our laboratory.[4] This set consisted of 66 active and 19 inactive compounds. The *literature set* (**Table B in S1 File**) was based on data obtained by others in various studies and contained chemically diverse compounds (26 actives, 65 inactives).[1,31,32,33,34,35] Compounds reported as inactive on hTAS2R39 were only included in the *literature set*, if they were tested at sufficiently high maximal concentrations (≥ 500 μM) in order to be in the same order of magnitude as inactive compounds from the *lab set*. The *blockers* included three recently discovered compounds, which reduced or eliminated activation of hTAS2R39 by receptor agonists.[6]

All compounds were prepared with MOE software from CCG (version 2012.10).[36] The 3D structures of the molecules were generated, partial charges (Gasteiger PEOE) were assigned, and the database energy minimization protocol with force field MMFF94x was used to enforce low energy conformations of the molecules. For the pharmacophore validation, multiple conformations of the compounds were needed, which can be subsequently fitted to the pharmacophore model. The conformational search was performed with a stochastic search (Rejection Limit 100, Iteration Limit 1000, RMS Gradient 0.005, MM Iteration Limit 200, Conformation Limit 200).

### Feature selection

In order to select the features that contribute most to the recognition of agonists from the lab and literature set, the number of true positives (TP), false positives (FP), true negatives (TN), and false negatives (FN) were calculated per pharmacophore validation. In addition, the recall (recall = TP/(TP+FN)), precision (precision = TP/(TP+FP)) rates and the Matthews correlation coefficient (MCC) (Equation 1) were calculated. The MCC ranges from -1 (no correlation) to 1 (full correlation).

$$\text{MCC=}\frac{\text{(TP×TN-FP×FN)}}{\sqrt{\text{((TP+FP)(TP+FN)(TN+FP)(TN+FN))}}}$$ (Equation 1)

## Results and Discussion

### Definition of bitter receptor binding pocket

**Fig 1:**
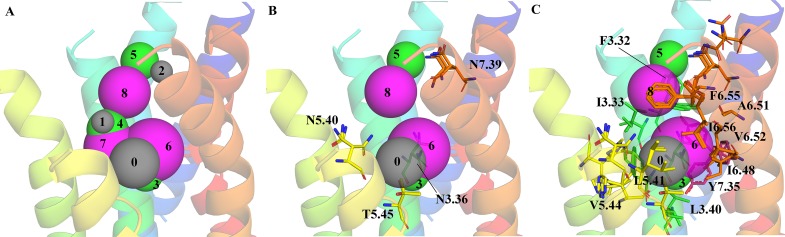
Homology model of the TM domains of hTAS2R39. TM I is depicted in dark blue, TM II in light blue, TM III in cyan, TM IV in light green, TM V in yellow, TM VI in orange, and TM VII in red. **A)** The Snooker pharmacophore hypothesis consists of acceptor features (numbers **0**, **1**, and **2** in gray), donor features (numbers **3**, **4**, and **5** in green), and hydrophobic features (numbers **6**, **7**, and **8** in magenta). Residues contributing to **B)** acceptor and donor features (**0**, **3**, and **5**) and **C)** hydrophobic features (**6**, and **8**) of the best performing feature combination are shown as sticks. All common rotamers are shown.

For hTAS2R39, a structure based pharmacophore model was built (**Fig. 1A**) using only sequence conservation statistics and the structure of the transmembrane helices of a rhodopsin subfamily structure. The binding pocket is located at the external site of the transmembrane region. This finding is in line with the known GPCR crystal structures of the rhodopsin subfamily and it is also commonly accepted for the bitter taste receptors.[8,12,14,29,37] The binding pocket is located between TM III, TM V, TM VI and TM VII. The amino acids contributing to the binding site are listed in **Table 1**.

**Table 1:** Snooker pharmacophore features. Feature type, feature number, radius and residue contributions by amino acid number.

Feature	Feature number	Radius [Å]	Residue number and contribution to feature
Acceptor	0	1.80	T5.45, 1.00
Acceptor	1	0.97	N5.40, 0.88; H4.56, 0.13
Acceptor	2	0.98	N7.39, 0.75; Q7.35, 0.25
Donor	3	1.82	T5.45, 0.81; N3.36, 0.13; N5.40, 0.06
Donor	4	2.16	N5.40, 0.82; H4.56, 0.18
Donor	5	2.10	N7.39, 1.00
Hydrophobic	6	3.32	V6.52, 0.28; I6.48, 0.16; L3.40, 0.15; L5.41, 0.11; F6.55, 0.10; F3.37, 0.06; V5.44, 0.05; I6.56, 0.04; F3.32, 0.03; A6.51, 0.01; Y7.45, 0.01
Hydrophobic	7	2.84	V5.44, 0.37; I3.33, 0.28; F3.37, 0.17; L5.41, 0.10; F6.55, 0.06; L3.40, 0.01
Hydrophobic	8	2.75	F6.55, 0.45; I3.33, 0.43; L5.41, 0.09; F3.32, 0.04

### Pharmacophore model of the binding site

Based on the residues in the receptor binding site, the pharmacophore features were calculated in Snooker. The calculation of the features was based on step (3) rotamer sampling and step (5) residue scoring of the Snooker methodology.

All common rotamers for alpha-helix residues (**Fig. 1B,C**) were considered and the density of interaction features surrounding a rotamer was weighted according to their true existence in alpha-helices of experimentally derived structures. In addition, the Snooker residue scoring had influence on the density of the interaction feature placement. The residue scoring in Snooker is based on the assumption that residues involved in ligand binding are conserved within a subfamily. For residues with a high conservation score the density of interaction features is larger than for residues with a lower score.

The interaction features obtained were subsequently translated into a pharmacophore hypothesis (**Fig. 1A**). For each feature type (acceptor, donor and hydrophobic) the top three features were generated. A list with the contribution of the amino acid residues to each feature can be found in Table 1. The hydrogen bond acceptor and donor features were assigned to only a few residues in the binding site (**Fig. 1B**, Table 1). The hydrophobic features of the pharmacophore model were based on up to 11 residues and had a radius of 2.75–3.32 Å (**Fig. 1C**, **Table 1**). These features seemed to describe the overall shape of the compounds that can bind to the receptor. Besides hydrophobic interactions, aromatic residues (F3.32, F6.55) could interact with the compounds via π-stacking. This is in line with the nature of protein-ligand interactions. Hydrogen bonds are very specific and occur at specific distances and angles, as a result of which acceptor and donor features comprise 1–3 residues. Hydrophobic interactions are less defined in their shape and direction, leading to larger hydrophobic features with 4–11 residues.

### Pharmacophore validation

Considering Snooker applications in the past, the binding site can be best described by 5–6 features. For the pharmacophore validation, all feature combinations with 5 out of 9 features or 6 out of 9 features were used. If all 9 features were to be mapped, the number of hits would be limited to only a few compounds, most likely many other compounds with potential for unravelling the hTAS2R39 binding pocket would have been missed. As measure for the quality of the pharmacophore validation recall, precision and the Matthews correlation coefficient (MCC) were calculated. All results which had a recall of more than 50% (Table 2, **Tables C-E in S1 File**) were further evaluated.

**Table 2:** Best pharmacophore validation results per set and 5 or 6 feature combination.

Compound set	Feature set	Recall	Precision	MCC
lab set	[0, 3, 6, 7, 8]	0.91	0.89	0.51
	[0, 3, 5, 6, 7, 8]	0.73	0.96	0.48
literature set	[0, 1, 6, 7, 8]	0.69	0.74	0.61
	[0, 1, 4, 6, 7, 8]	0.58	0.87	0.35
combined set	[0, 3, 5, 6, 8]	0.63	0.97	0.44
	[0, 3, 5, 6, 7, 8]	0.61	0.97	0.44

**Fig 2:**
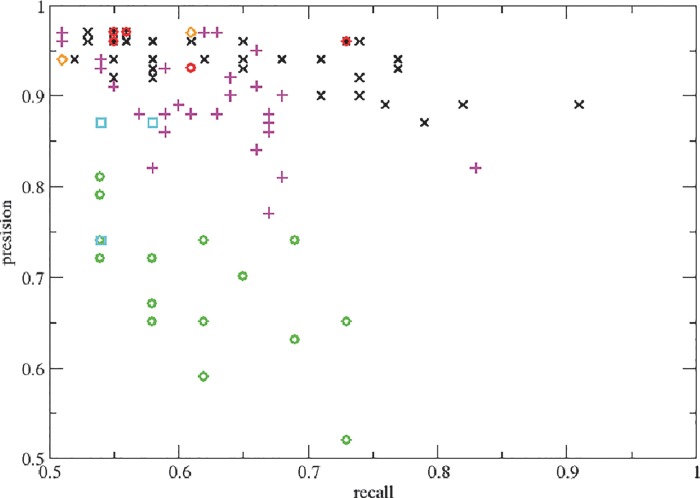
Pharmacophore results plot. 5 feature pharmacophore lab set (black x), 5 feature pharmacophore literature set (green circle), 5 featured combined set (magenta plus), 6 feature pharmacophore lab set (red circle), 6 feature pharmacophore literature set (cyan box) and 6 featured combined set (orange diamond).

Comparing the results of 5 and 6 feature pharmacophores, it turned out that the overall performance was better for the 5 featured pharmacophores (**Fig. 2**, **Table 2**). The 6 feature pharmacophores are probably too specific to certain ligand classes (and restrict ligands too much), whereas the 5 feature pharmacophores capture the key binding modes, while still allowing for some variation in the ligands.

Based on the better results for 5 feature pharmacophores, we analyzed these in more detail. The best performing set of features for the lab set (**Table 2**) was [0, 3, 6, 7, 8] (recall: 91%, precision: 89%, MCC: 0.51) and for the literature set (**Table 2**) was [0, 1, 6, 7, 8] (recall: 69%, precision: 74%, MCC: 0.61). Both pharmacophores were similar and shared 4 of the 5 features. It is known that different ligands often share a core of ligand-binding amino acid residues, but non-overlapping binding features occur as well.

**Fig 3:**
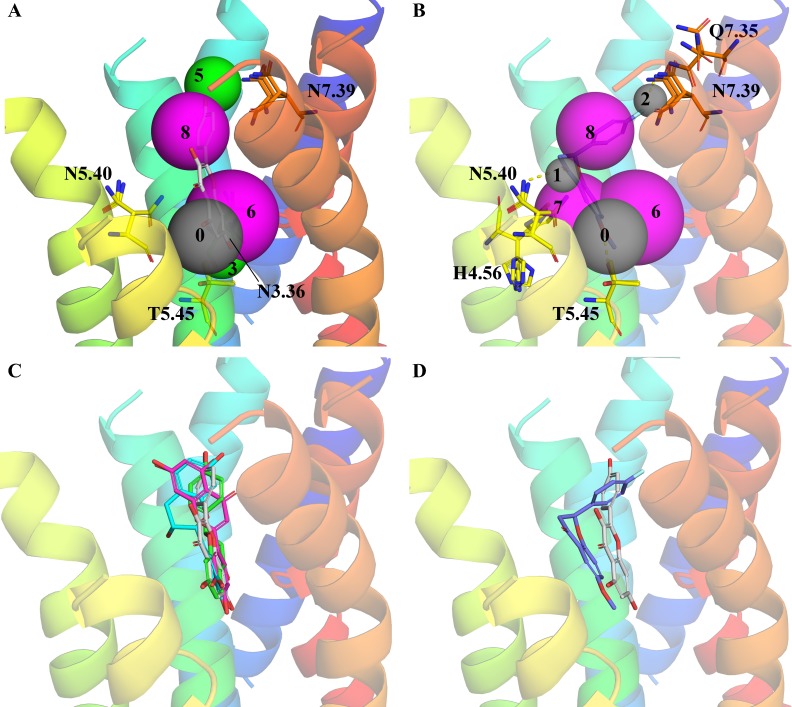
Selection of compounds fitted into the pharmacophore. **A)** Fitting of agonist kaempferol (gray) into the pharmacophore features **0**, **3**, **5**, **6**, **8**. Residues, which make hydrogen bonds to the agonist, are shown as sticks. **B)** Fitting of the blocker 4’-fluoro-6-methoxyflavanone (S-enantiomer, blue) into the pharmacophore features **0**, **1**, **2**, **7**, **8**. Residues, which make hydrogen bonds (yellow dashes) to the blocker, are shown as sticks. **C)** Fitting of kaempferol (gray), luteolin (pink), naringenin (green), and epicatechin (cyan). **D)** Fitting of the kaempferol (gray) and 4’-fluoro-6-methoxyflavanone (blue). The structures of 4’-fluoro-6-methoxyflavanone, luteolin, naringenin, and epicatechin are shown in **Fig. 4**.

Combining both the lab and literature set (Table 2) allowed determination of the feature combination, giving the best validation results. Best validation results were obtained for feature combination [0, 3, 5, 6, 8] (recall: 63%, precision: 97%, MCC: 0.44) (Fig. 2). Based on the combined pharmacophore validation results, the features most selective in compound recognition were features 0, 3 and 5. Hydrogen bond acceptor feature 0 refers to residue T5.45, hydrogen bond donor feature 3 refers to residue T5.45, N3.36 and N5.40 and hydrogen bond donor feature 5 refers to N7.39. With 81%, T5.45 (Table 1) contributed most to feature 3, although N3.36 and N5.40 contributed as well. This resulted in the location of feature 3 at the bottom of the binding site. Without the contribution of N3.36 and N5.40, feature 3 and feature 0 would coincide (**Fig. 1B** and **3A**). Feature 5 was located at the top of the binding site.

GPCRs which have been co-crystalized with agonists show that deep in the pocket between TM III, IV, V, VI, and VII the activation trigger is positioned. One example is the activation of the beta-1 adrenergic receptor (b1-AR), triggered by the interaction to S5.42 in TM V.[37] In the case of the Snooker hTAS2R39 model, T5.45 is located at this position (the numbering is shifted by 3 positions, as the beginning of helix V was adjusted, see methods) and might be the key residue for receptor activation. Interestingly, there are also similarities in key amino acids for ligand recognition found with our model and those in two other bitter taste receptors. Behrens et al. mentioned the importance of N3.36, which is probably involved in binding of agonists to hTAS2R16, -30, -38 and -46, and of residues close to N3.36 (L3.32, L3.33 and E3.37), which are involved in binding to hTAS2R1.[38] Furthermore, it was proposed that W3.32, A3.33, N3.36, H3.37, and N3.40 were key residues for ligand interaction with hTAS2R31, -43, and -46,[18] and S3.29, W3.32, V3.33, N3.36, Q5.40, and L5.43 for ligand interaction with hTAS2R10.[10] Although there is striking similarity in key residues (or TM regions) involved in ligand binding in hTAS2R39 and other hTAS2Rs, the residues do not need to be identical, as has been demonstrated for GPCRs of the rhodopsin subfamily, where different amino acids were important for ligand binding and activation in various representatives.[26]

### Comparison of structure-based pharmacophore model to ligand-based pharmacophore model

In a previous study, ligand-based pharmacophore models were developed for isoflavonoids and flavonoids with respect to hTAS2R39.[4] The best results for the ligand-based model were achieved for a 6-feature pharmacophore, which allowed the ligands to map 5 of 6 features and comprised of three hydrogen bond donor sites, one hydrogen bond acceptor site, and two aromatic ring structures, of which one had to be hydrophobic.

Comparison of ligand-based and structure-based pharmacophore models revealed that the results were similar and ligand-based and structure-based models overlapped regarding the occurrence of the following 5 features: 1 hydrogen bond acceptor (feature 0), 2 hydrogen bond donors (feature 3, 5), and 2 hydrophobic features (feature 6, 8). In case of the ligand-based model, better results were obtained with in total three donor features and the possibility to miss one of the six features, and in case of the structure-based model, better results were obtained with in total three hydrophobic features.

Regarding size, both models were comparable as well. The distance between the two hydrophobic features in the ligand-based model was 6.8 Å, and in the structure-based model 6.7 Å. The two features furthest apart in the ligand-based model were two hydrogen bond donors with a distance of 10.7 Å. In the structure-based model the two features furthest apart were 3 and 5 with a distance of 12.8 Å.

Comparison of the best models for the lab set revealed that both approaches led to very good results. The performance of the ligand-based model (recall: 86%, precision: 95%, MCC: 0.57) was even slightly better than that of the structure-based model (recall: 91%, precision: 89%, MCC: 0.51). Nevertheless, the ligand-based model was limited to the use for isoflavonoids, flavonoids and structurally closely related compounds. The structure-based model offers broader applicability to structurally diverse bitter receptor agonists. Furthermore, the structure-based model can account for two binding modes, agonist and antagonist (or blocker), together with their respective interacting amino acids, as will be described below.

### Blocker binding

**Fig 4:**
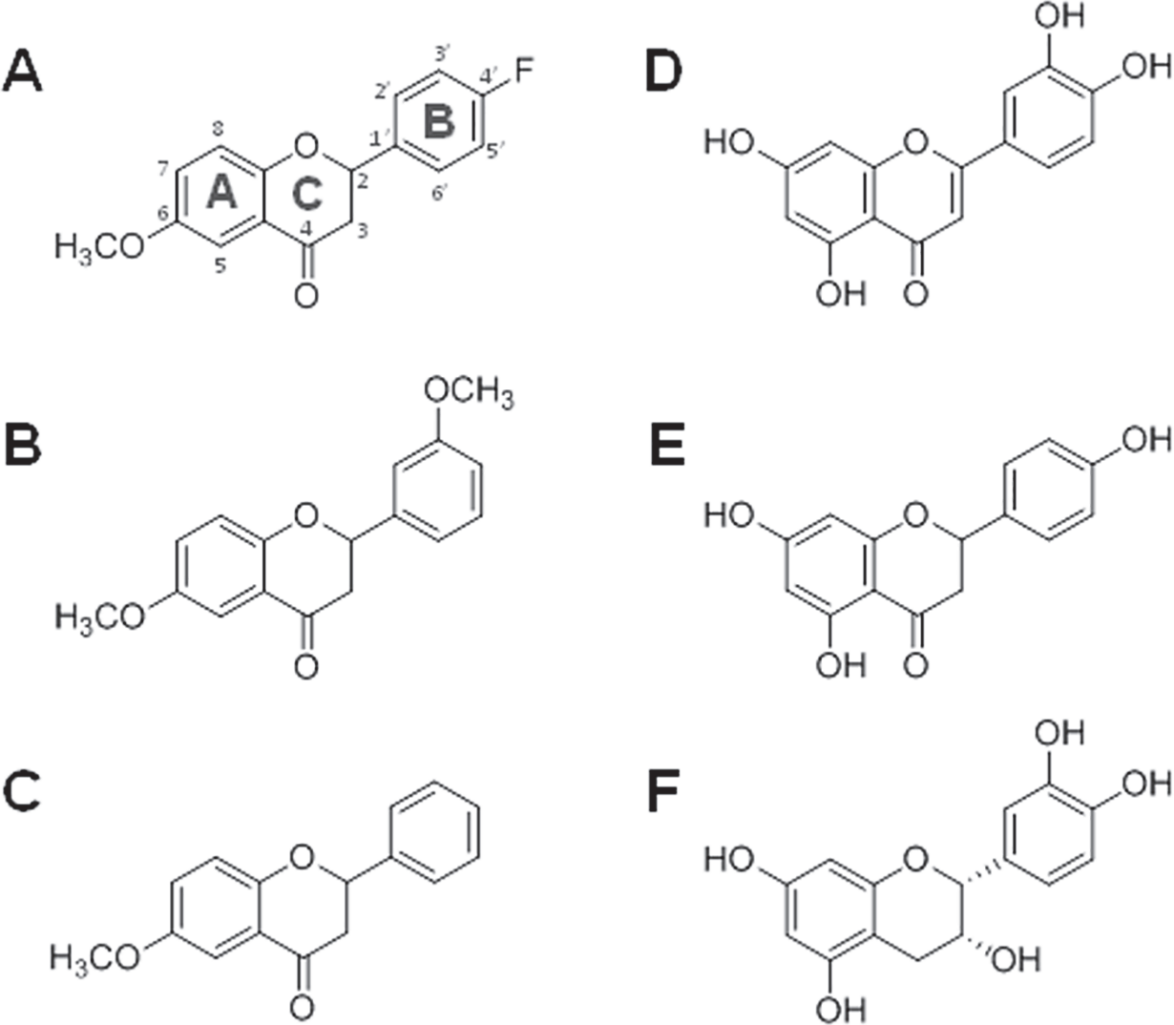
Structures of hTAS2R39 blockers (A-C) and agonists (D-F). 4’-fluoro-6-methoxyflavanone (**A**), 6,3’-dimethoxyflavanone (**B**), 6-methoxyflavanone (**C**), luteolin (**D**), naringenin (**E**), and epicatechin (**F**). Flavonoid-specific ring nomenclature (A/B/C-ring) and the atom numbers are illustrated in (**A**).

The three compounds 4’-fluoro-6-methoxyflavanone, 6,3’-dimethoxyflavanone, and 6-methoxyflavanone (in decreasing order of potency, depicted in Fig. 4) have been reported as blockers of hTAS2R39.[6] These compounds decreased or eliminated the activation of hTAS2R39 by several agonists, suggesting that they bind to the receptor without activating it. The blocker molecules are characterized by lacking a double bond in the C-ring between atoms 2 and 3 (subclass ‘flavanones’). This results in a tetrahedral conformation at atom 2 for the blockers compared to most agonists, which are planar at this position (**Fig. 3B,C**). Besides lacking the C-ring double bond, it is important to note that flavanones were only functional as blockers when they possessed a methoxy group at atom 6 (e.g. a methoxy group at atom 7 or a hydroxyl group at atom 6 did not result in blocking).

With respect to the results of the 5 and 6 features pharmacophore validation (**Table F in S1 File**), all blockers could be fitted into the pharmacophore model containing up to three hydrogen bond acceptor features and three hydrophobic features. In general, the *S*-enantiomer could more often be fitted into the pharmacophore model than the *R*-enantiomer. However, it could not be verified whether the *R*-enantiomer is inactive, as a racemic mixture of 4’-fluoro-6-methoxyflavanone, 6,3’-dimethoxyflavanone, and 6-methoxyflavanone was used in the receptor assay.

Agonists and blockers share fitting to feature 0, which indicates an interaction to T5.45 and feature 6 and 8, which fits the hydrophobic core of the molecules. Due to the stereocenter introduced, the blockers were placed crooked into the pharmacophore and not linear as most of the agonists (**Fig. 3B**). Reviewing the properties of the blockers showed that they had no hydrogen bond donor. This was in contrast to all hTAS2R39 agonists, which comprised at least one hydrogen bond donor (with the exception of two compounds, flavone and xanthone). Lacking a hydrogen bond donor, the blockers did not map to feature 3, which seemed important for receptor activation. As displayed in **Fig. 3B**, the hydrogen bond acceptor groups of the blocker fitted optimally into the hydrogen bond acceptor features of the pharmacophore model with features [0, 1, 2, 7, 8]. This positioning allowed 4’-fluoro-6-methoxyflavanone and 6,3’-dimethoxyflavanone to make a hydrogen bond to Q7.35 and/or N7.39 (**Fig. 3B**). Moreover, the blockers could interact with the side chain of F6.55 by forming hydrophobic interactions (not shown in Fig. 3B). These interactions could explain the different strength of the blockers (fluoro > methoxy > hydrogen). If both interactions occurred, hydrophobic interaction with F6.55 and hydrogen bonding with Q7.35 / N7.39, the blocker appeared to be more potent than if only one interaction was possible. In addition, the hydrogen bond of 4’-fluoro-6-methoxyflavanone is stronger and in a better angle to the protein than the one of 6,3’-dimethoxyflavanone.

Considering flavonoids, the difference between agonists and blockers is determined by planarity of the ring structures, in combination with the occurrence of hydrogen bond donors. **Fig. 3C** shows kaempferol, luteolin, naringenin, and epicatechin, compounds with excellent agonistic properties on hTAS2R39.[4] Thus, not all non-linear molecules are blockers, as e.g. flavanols are hTAS2R39 agonists, due to their hydrogen bond donor groups. The absence of hydrogen bond donors in combination with a crooked position of the molecule in the binding site are prerequisites for blocking properties on hTAS2R39 (**Fig. 3D**).

For rhodopsin subfamily GPCRs, it is known that the TM domains around the extracellular ligand binding site are tightened up upon agonist binding, which goes along with a conformational change of the TM domains on the intracellular side. These conformational changes are important for receptor activation.[37] Upon blocker binding to rhodopsin subfamily GPCRs, the contraction of the TM domains in the binding site is sterically hindered. For the taste receptors a similar conformational change for receptor activation might be expected. Based on the structure-based pharmacophore model of hTAS2R39, we can propose that the blockers studied behave very similar to rhodopsin subfamily blockers. The crooked binding mode of the blockers, which is stabilized by the three (or less) hydrogen bonds (**Fig. 3B**) to the receptor, can hinder the conformational change in the TM domains, which is needed for receptor activation.

## Conclusion

With the structure-based pharmacophore model derived with Snooker details about receptor binding sites at a molecular level can be proposed, which are in accordance to amino acids mentioned in related studies. Furthermore, the structure-based pharmacophore model gives insight into the differences of agonist and blocker binding. The structure-based model has been validated with large sets of previously published compounds (two large sets of agonists and inactive compounds, and one small set of blockers, as only few blockers are known). As these compounds were not used to build the model, they are suited as *retro* perspective experimental validation of the structure-based pharmacophore model. Our structure-based pharmacophores overlap with previously generated ligand-based pharmacophores, suggesting that we have indeed successfully identified the key interaction features of the hTAS2R39 receptor. This enabled us to generate a pose of each hTAS2R39 compound and optimize these by optimizing the interactions. Our pharmacophore model shows that flavonoid-derived blockers bind differently to the receptor than (iso)flavonoid-based agonists. Due to the tetrahedral conformation of the C-ring carbons 2 and 3, a crooked position of the molecule in the binding site is forced. In combination with the absence of hydrogen bond donors, this geometry leads to blocking properties, the strength of which is influenced by interaction with amino acid residues on the upper side of the binding pocket. Prospective validation of the model is desirable but not mandatory to ensure the quality of the derived model, as the compounds were not used to build the model. To verify all hypotheses about ligand binding and activation, complex site-directed mutagenesis studies would have to be conducted which would include the different use of ligand binding residues by the different ligands and the potentially different activation mechanism.

## Supporting Information

S1 File Table A in S1 File Compounds lab set. Table B in S1 File Compounds literature set. **Table C in S1 File** Results pharmacophore screening lab set. **Table D in S1 File** Results pharmacophore screening literature set. **Table E in S1 File** Results pharmacophore screening in which lab and literature sets are combined. **Table F in S1 File** Results list of pharmacophore screening with the blocker set. **Fig. A in S1 File Multiple sequence alignment tree.**
(PDF)

S2 File Alignment_012.fasta: Bitter taste receptor alignment as obtained from GPCRDB with species information. (FASTA)

S3 File Pharmacophore.txt: Pharmacophore coordinates. (TXT)

S4 File T2R39_MODEL.mol2: Protein coordinates. (MOL2)

## References

[1] Meyerhof W, Batram C, Kuhn C, Brockhoff A, Chudoba E, et al (2010) The molecular receptive ranges of human TAS2R bitter taste receptors. Chem. Senses 35: 157–170. 10.1093/chemse/bjp092 20022913

[2] Thalmann S, Behrens M, Meyerhof W (2013) Major haplotypes of the human bitter taste receptor TAS2R41 encode functional receptors for chloramphenicol. Biochem. Biophys. Res. Commun. 435: 267–273. 10.1016/j.bbrc.2013.04.066 23632330

[3] Roland WS, Vincken J-P, Gouka RJ, van Buren L, Gruppen H, et al (2011) Soy isoflavones and other isoflavonoids activate the human bitter taste receptors hTAS2R14 and hTAS2R39. J. Agric. Food Chem. 59: 11764–11771. 10.1021/jf202816u 21942422

[4] Roland WS, van Buren L, Gruppen H, Driesse M, Gouka RJ, et al (2013) Bitter taste receptor activation by flavonoids and isoflavonoids: modeled structural requirements for activation of hTAS2R14 and hTAS2R39. J. Agric. Food Chem. 61: 10454–10466. 10.1021/jf403387p 24117141

[5] Bohin MC, Roland WS, Gruppen H, Gouka RJ, van der Hijden HT, et al (2013) Evaluation of the bitter-masking potential of food proteins for EGCG by a cell-based human bitter taste receptor assay and binding studies. J. Agric. Food Chem. 61: 10010–10017. 10.1021/jf4030823 24093533

[6] Roland WS, Gouka RJ, Gruppen H, Driesse M, van Buren L, et al (2014) 6-Methoxyflavanones as Bitter Taste Receptor Blockers for hTAS2R39. PLoS ONE 9: e94451 10.1371/journal.pone.0094451 24722342 PMC3983201

[7] Adler E, Hoon MA, Mueller KL, Chandrashekar J, Ryba NJ, et al (2000) A novel family of mammalian taste receptors. Cell 100: 693–702. 10761934 10.1016/s0092-8674(00)80705-9

[8] Upadhyaya J, Pydi SP, Singh N, Aluko RE, Chelikani P (2010) Bitter taste receptor T2R1 is activated by dipeptides and tripeptides. Biochem. Biophys. Res. Commun. 398: 331–335. 10.1016/j.bbrc.2010.06.097 20599705

[9] Dai W, You Z, Zhou H, Zhang J, Hu Y (2011) Structure-function relationships of the human bitter taste receptor hTAS2R1: insights from molecular modeling studies. J. Recept. Signal Transduct. Res. 31: 229–240. 10.3109/10799893.2011.578141 21619450

[10] Born S, Levit A, Niv MY, Meyerhof W, Behrens M (2013) The human bitter taste receptor TAS2R10 is tailored to accommodate numerous diverse ligands. J. Neurosci. 33: 201–213. 10.1523/JNEUROSCI.3248-12.2013 23283334 PMC6618634

[11] Miguet L, Zhang Z, Grigorov MG (2006) Computational studies of ligand-receptor interactions in bitter taste receptors. J. Recept. Signal Transduct. Res. 26: 611–630. 17118801 10.1080/10799890600928210

[12] Sakurai T, Misaka T, Ishiguro M, Masuda K, Sugawara T, et al (2010) Characterization of the beta-D-glucopyranoside binding site of the human bitter taste receptor hTAS2R16. J. Biol. Chem. 285: 28373–28378. 10.1074/jbc.M110.144444 20605788 PMC2934701

[13] Slack JP, Brockhoff A, Batram C, Menzel S, Sonnabend C, et al (2010) Modulation of bitter taste perception by a small molecule hTAS2R antagonist. Curr. Biol. 20: 1104–1109. 10.1016/j.cub.2010.04.043 20537538 PMC2925244

[14] Floriano WB, Hall S, Vaidehi N, Kim U, Drayna D, et al (2006) Modeling the human PTC bitter-taste receptor interactions with bitter tastants. J. Mol. Model. 12: 931–941. 16607493 10.1007/s00894-006-0102-6

[15] Biarnes X, Marchiori A, Giorgetti A, Lanzara C, Gasparini P, et al (2010) Insights into the binding of Phenyltiocarbamide (PTC) agonist to its target human TAS2R38 bitter receptor. PLoS ONE 5: e12394 10.1371/journal.pone.0012394 20811630 PMC2928277

[16] Tan J, Abrol R, Trzaskowski B, Goddard WA 3rd (2012) 3D structure prediction of TAS2R38 bitter receptors bound to agonists phenylthiocarbamide (PTC) and 6-n-propylthiouracil (PROP). J. Chem. Inf. Model. 52: 1875–1885. 10.1021/ci300133a 22656649

[17] Marchiori A, Capece L, Giorgetti A, Gasparini P, Behrens M, et al (2013) Coarse-grained/molecular mechanics of the TAS2R38 bitter taste receptor: experimentally-validated detailed structural prediction of agonist binding. PLoS ONE 8: e64675 10.1371/journal.pone.0064675 23741366 PMC3669430

[18] Brockhoff A, Behrens M, Niv MY, Meyerhof W (2010) Structural requirements of bitter taste receptor activation. Proc. Natl. Acad. Sci. USA 107: 11110–11115. 10.1073/pnas.0913862107 20534469 PMC2890741

[19] Levit A, Barak D, Behrens M, Meyerhof W, Niv MY (2012) Homology model-assisted elucidation of binding sites in GPCRs. Methods Mol. Biol. 914: 179–205. 10.1007/978-1-62703-023-6_11 22976029

[20] Behrens M, Meyerhof W (2013) Bitter taste receptor research comes of age: from characterization to modulation of TAS2Rs. Semin. Cell Dev. Biol. 24: 215–221. 10.1016/j.semcdb.2012.08.006 22947915

[21] Ley JP, Dessoy M, Paetz S, Blings M, Hoffmann-Lucke P, et al (2012) Identification of enterodiol as a masker for caffeine bitterness by using a pharmacophore model based on structural analogues of homoeriodictyol. J. Agric. Food Chem. 60: 6303–6311. 10.1021/jf301335z 22670770

[22] Fletcher JN, Kinghorn AD, Slack JP, McCluskey TS, Odley A, et al (2011) *In vitro* evaluation of flavonoids from Eriodictyon californicum for antagonist activity against the bitterness receptor hTAS2R31. J. Agric. Food Chem. 59: 13117–13121. 10.1021/jf204359q 22059530 PMC4391372

[23] Brockhoff A, Behrens M, Roudnitzky N, Appendino G, Avonto C, et al (2011) Receptor agonism and antagonism of dietary bitter compounds. J. Neurosci. 31: 14775–14782. 10.1523/JNEUROSCI.2923-11.2011 21994393 PMC6703385

[24] Greene TA, Alarcon S, Thomas A, Berdougo E, Doranz BJ, et al (2011) Probenecid inhibits the human bitter taste receptor TAS2R16 and suppresses bitter perception of salicin. PLoS ONE 6: e20123 10.1371/journal.pone.0020123 21629661 PMC3101243

[25] Sanders MP, Verhoeven S, de Graaf C, Roumen L, Vroling B, et al (2011) Snooker: a structure-based pharmacophore generation tool applied to class A GPCRs. J. Chem. Inf. Model. 51: 2277–2292. 10.1021/ci200088d 21866955

[26] Sanders MP, Fleuren WW, Verhoeven S, van den Beld S, Alkema W, et al (2011) ss-TEA: Entropy based identification of receptor specific ligand binding residues from a multiple sequence alignment of class A GPCRs. BMC Bioinformatics. 12: 332 10.1186/1471-2105-12-332 21831265 PMC3162937

[27] Vroling B, Sanders M, Baakman C, Borrmann A, Verhoeven S, et al (2011) GPCRDB: information system for G protein-coupled receptors. Nucleic Acids Res. 39: D309–319. 10.1093/nar/gkq1009 21045054 PMC3013641

[28] Singh N, Pydi SP, Upadhyaya J, Chelikani P (2011) Structural basis of activation of bitter taste receptor T2R1 and comparison with Class A G-protein-coupled receptors (GPCRs). J. Biol. Chem. 286: 36032–36041. 10.1074/jbc.M111.246983 21852241 PMC3195589

[29] Li J, Edwards PC, Burghammer M, Villa C, Schertler GF (2004) Structure of bovine rhodopsin in a trigonal crystal form. J. Mol. Biol. 343: 1409–1438. 15491621 10.1016/j.jmb.2004.08.090

[30] Renner S, Schneider G (2004) Fuzzy pharmacophore models from molecular alignments for correlation-vector-based virtual screening. J. Med. Chem. 47: 4653–4664. 15341481 10.1021/jm031139y

[31] Narukawa M, Noga C, Ueno Y, Sato T, Misaka T, et al (2011) Evaluation of the bitterness of green tea catechins by a cell-based assay with the human bitter taste receptor hTAS2R39. Biochem. Biophys. Res. Commun. 405: 620–625. 10.1016/j.bbrc.2011.01.079 21272567

[32] Ueno Y, Sakurai T, Okada S, Abe K, Misaka T (2011) Human bitter taste receptors hTAS2R8 and hTAS2R39 with differential functions to recognize bitter peptides. Biosci. Biotechnol. Biochem. 75: 1188–1190. 21670512 10.1271/bbb.100893

[33] Kohl S, Behrens M, Dunkel A, Hofmann T, Meyerhof W (2013) Amino acids and peptides activate at least five members of the human bitter taste receptor family. J. Agric. Food Chem. 61: 53–60. 10.1021/jf303146h 23214402

[34] Soares S, Kohl S, Thalmann S, Mateus N, Meyerhof W, et al (2013) Different phenolic compounds activate distinct human bitter taste receptors. J. Agric. Food Chem. 61: 1525–1533. 10.1021/jf304198k 23311874

[35] Hellfritsch C, Brockhoff A, Stahler F, Meyerhof W, Hofmann T (2012) Human psychometric and taste receptor responses to steviol glycosides. J. Agric. Food Chem. 60: 6782–6793. 10.1021/jf301297n 22616809

[36] ) Molecular Operating Environment (MOE), 2011.10. Chemical Computing Group Inc, 1010 Sherbooke St West, Suite #910, Montreal, QC, Canada, H3A 2R7.

[37] Lebon G, Warne T, Edwards PC, Bennett K, Langmead CJ, et al (2011) Agonist-bound adenosine A2A receptor structures reveal common features of GPCR activation. Nature 474: 521–525. 10.1038/nature10136 21593763 PMC3146096

[38] Behrens M, Gunn HC, Ramos PC, Meyerhof W, Wooding SP (2013) Genetic, functional, and phenotypic diversity in TAS2R38-mediated bitter taste perception. Chem. Senses 38: 475–484. 10.1093/chemse/bjt016 23632915

