# Supplementary material for: Snooker Structure-Based Pharmacophore Model Explains Differences in Agonist and Blocker Binding to Bitter Receptor hTAS2R39
Source: PLoS One. 2015 Mar 2;10(3):e0118200. doi: 10.1371/journal.pone.0118200 (PMC4346584; doi:10.1371/journal.pone.0118200)
Supplement: S1 File — Table C in S1 File Results pharmacophore screening lab set. Table D in S1 File Results pharmacophore screening literature set. Table E in S1 File Results pharmacophore screening in which lab and literature sets are combined. Table F in S1 File Results list of pharmacophore screening with the blocker set. Fig. A in S1 File Multiple sequence alignment tree. (PDF) [file pone.0118200.s001.pdf]

Snooker structure-based pharmacophore model explains differences in agonist and blocker binding to bitter receptor hTAS2R39

Wibke S.U. Roland[1,2], Marijn P.A. Sanders[3,4], Leo van Buren[5], Robin J Gouka[5], Harry Gruppen[1], Jean-Paul Vincken[1], Tina Ritschel[3,*]

Supporting Information

Table A in S1 file Compounds lab set

Name	Agonist?
Acetylgenistin	yes
Apigenin	yes
Biochanin A	yes
Butein	yes
(+)-Catechin	yes
Chrysin	yes
Coumestrol	yes
Cyanidin chloride	yes
Daidzein	yes
Datiscetin	yes
3,2'-Dihydroxychalcone	yes
5,2'-Dihydroxyflavone	yes
5,4'-Dihydroxyflavone	yes
6,4'-Dihydroxyflavone	yes
7,4'-Dihydroxyflavone	yes
5,7-Dimethoxyflavone	yes
Equol	yes
Eriodictyol	yes
Eriodictyolchalcone	yes
Fisetin	yes
Flavone	yes
Formononetin	yes
Fustin	yes
Genistein	yes
Genistin	yes
Genkwanin	yes
Glycitein	yes
Glycitin	yes
Gossypetin	yes

Herbacetin	yes
Hesperetin	yes
Homoeriodictyol	yes
5-Hydroxyflavone	yes
7-Hydroxyisoflavone	yes
4'-Hydroxyflavone	yes
4'-Hydroxy-7-methoxyflavone	yes
Isoliquiritigenin	yes
Isorhamnetin	yes
Kaempferol	yes
Liquiritigenin	yes
Luteolin	yes
Malonylgenistin	yes
6-Methoxyluteolin	yes
Morin	yes
Myricetin	yes
Naringenin	yes
Pelargonidin chloride	yes
Phloretin	yes
Pinocembrin	yes
Quercetagetin	yes
Resveratrol	yes
Scutellarein	yes
Silibinin	yes
Sulfuretin	yes
(+)-Taxifolin	yes
3,6,3',4'-Tetrahydroxyflavone	yes
Tricetin	yes
2,2',4'-Trihydroxychalone	yes
4,2',5'-Trihydroxychalcone	yes
3,7,4'-Trihydroxyflavone	yes
7,3',4'-Trihydroxyflavone	yes
5,7,2'-Trihydroxyflavone	yes
6,7,4'-Trihydroxyisoflavone	yes
7,8,4'- Trihydroxyisoflavone	yes
7,3',4'-Trihydroxyisoflavone	yes
Xanthone	yes
Apigeninidin chloride	no
Chalcone	no
Chrysoeriol	no
Daidzin	no
4,2'-Dihydroxychalcone	no
5,3'-Dihydroxyflavone	no
6,7-Dimethoxyflavone	no
7,4'-Dimethoxy-5-hydroxyisoflavone	no

7,4'-Dimethoxyisoflavone	no
Flavanone	no
Flavonol	no
Gardenin A	no
4'-Hydroxyflavanone	no
5-Hydroxy-3'-methoxyflavone	no
4'-Hydroxy-6-methoxyflavone	no
Isoflavone	no
6-Methoxyflavanone	no
6-Methoxyflavone	no
5,7,4'-Trimethoxyflavone	no

Table B in S1 file Compounds literature set

Name	Literature	Agonist?
Acetaminophen	Meyerhof et al. 2010	yes
Amarogentin	Meyerhof et al. 2010	yes
Azathioprine	Meyerhof et al. 2010	yes
Chloramphenicol	Meyerhof et al. 2010	yes
Chloroquine	Meyerhof et al. 2010	yes
Chlorpheniramine	Meyerhof et al. 2010	yes
Colchicine	Meyerhof et al. 2010	yes
Denatonium benzoate	Meyerhof et al. 2010	yes
Diphenidol	Meyerhof et al. 2010	yes
(-)-Epicatechin	Narukawa et al. 2011, Roland et al. 2013, Soares et al. 2013	yes
(-)-Epicatechin gallate	Narukawa et al. 2011, Roland et al. 2013	yes
(-)-Epigallocatechin	Narukawa et al. 2011, Roland et al. 2013	yes
(-)-Epigallocatechin gallate	Narukawa et al. 2011, Roland et al. 2013	yes
3-(2-Hydroxyethyl)-indole	Kohl et al. 2013	yes
Ile-Phe	Kohl et al. 2013	yes
Leu-Trp	Kohl et al. 2013	yes
Leu-Val-Tyr-Pro-Phe-Pro-Gly-Pro-Ile-His-Asn	Kohl et al. 2013	yes
PGG	Soares at al. 2013	yes
Phe-Phe-Pro-Arg	Ueno et al. 2011	yes
Pro-Arg	Ueno et al. 2011	yes
Quinine	Meyerhof et al. 2010	yes
Thiamine	Meyerhof et al. 2010	yes
D-Trp	Kohl et al. 2013	yes
Trp-Trp	Kohl et al. 2013	yes
Trp-Trp-Trp	Kohl et al. 2013	yes
Tyr-Pro-Phe-Pro-Gly-Pro-Ile-His-Asn-Ser	Kohl et al. 2013	yes
Acesulfame K	Meyerhof et al. 2010	no
2-Acetylpyrazine	Meyerhof et al. 2010	no

3-Acetylindole	Kohl et al. 2013	no
L-Ala	Kohl et al. 2013	no
D-Amygdalin	Meyerhof et al. 2010	no
Arbutin	Meyerhof et al. 2010	no
L-Arg	Ueno et al. 2011, Kohl et al. 2013	no
L-Asn	Kohl et al. 2013	no
Campher	Meyerhof et al. 2010	no
Caprolactam	Meyerhof et al. 2010	no
Cromolyn	Meyerhof et al. 2010	no
Cycloheximid	Meyerhof et al. 2010	no
L-Cys	Kohl et al. 2013	no
Divinylsulfoxid	Meyerhof et al. 2010	no
Ethylpyrazine	Meyerhof et al. 2010	no
L-Gln	Kohl et al. 2013	no
L-Glu	Kohl et al. 2013	no
Gly	Kohl et al. 2013	no
Helicin	Meyerhof et al. 2010	no
L-His	Kohl et al. 2013	no
4-Hydroxyanisol	Meyerhof et al. 2010	no
L-Ile	Kohl et al. 2013	no
Indole	Kohl et al. 2013	no
Indole-3-acetamide	Kohl et al. 2013	no
Indole-3-acetic acid	Kohl et al. 2013	no
Indole-3-acrboxylic acid	Kohl et al. 2013	no
L-Leu	Kohl et al. 2013	no
Leu-Leu	Kohl et al. 2013	no
Leu-Leu-Leu	Kohl et al. 2013	no
L-Lys	Kohl et al. 2013	no
L-Met	Kohl et al. 2013	no
Methimazole	Meyerhof et al. 2010	no
Metronidazole	Meyerhof et al. 2010	no
N,N´-Ethylene thiourea	Meyerhof et al. 2010	no
N-Ethyltiourea	Meyerhof et al. 2010	no
Ouabain	Meyerhof et al. 2010	no
Oxolinic acid	Meyerhof et al. 2010	no
D-Phe	Kohl et al. 2013	no
L-Phe	Ueno et al. 2011, Kohl et al. 2013	no
Phe-Ile	Kohl et al. 2013	no
1,10-Phenanthroline	Meyerhof et al. 2010	no
L-Phe-L-Phe	Ueno et al. 2011	no
L-Phe-L-Pro	Ueno et al. 2011	no
Phe-Trp	Kohl et al. 2013	no
Picrotoxinin	Meyerhof et al. 2010	no
L-Pro	Ueno et al. 2011, Kohl et al. 2013	no
PROP	Meyerhof et al. 2010	no

RebaudiosideA	Hellfritsch et al. 2012	no
Saccharin	Meyerhof et al. 2010	no
D-Salicin	Meyerhof et al. 2010	no
L-Ser	Kohl et al. 2013	no
Serotonin	Kohl et al. 2013	no
Sinigrin	Meyerhof et al. 2010	no
Sodium benzoate	Meyerhof et al. 2010	no
Sodium cyclamate	Meyerhof et al. 2010	no
Sodium thiocyanate	Meyerhof et al. 2010	no
Stevioside	Hellfritsch et al. 2012	no
Taurine	Meyerhof et al. 2010	no
L-Thr	Kohl et al. 2013	no
L-Trp	Kohl et al. 2013	no
Trp-Leu	Kohl et al. 2013	no
Trp-Phe	Kohl et al. 2013	no
Trp-Pro	Kohl et al. 2013	no
L-Tyr	Kohl et al. 2013	no
L-Val	Kohl et al. 2013	no

Table C in S1 file Results pharmacophore screening lab set.

Feature set	Recall	Precision	MCC
[0, 3, 6, 7, 8]	0.91	0.89	0.51
[0, 3, 5, 6, 8]	0.74	0.96	0.50
[0, 3, 5, 6, 7]	0.77	0.94	0.49
[0, 3, 5, 7, 8]	0.73	0.96	0.48
[3, 5, 6, 7, 8]	0.77	0.93	0.45
[4, 5, 6, 7, 8]	0.71	0.94	0.43
[0, 3, 4, 6, 7]	0.65	0.96	0.41
[3, 4, 6, 7, 8]	0.68	0.94	0.40
[3, 4, 5, 6, 8]	0.56	0.97	0.38
[0, 4, 5, 6, 7]	0.61	0.96	0.37
[0, 5, 6, 7, 8]	0.82	0.89	0.37
[1, 3, 6, 7, 8]	0.74	0.92	0.37
[0, 1, 4, 6, 7]	0.65	0.94	0.37
[3, 4, 5, 6, 7]	0.55	0.97	0.37
[3, 4, 5, 7, 8]	0.53	0.97	0.36
[2, 3, 5, 6, 8]	0.58	0.96	0.35
[0, 2, 5, 6, 8]	0.58	0.96	0.35
[2, 5, 6, 7, 8]	0.62	0.94	0.34
[0, 4, 5, 7, 8]	056	0.96	0.34
[0, 1, 3, 6, 7]	0.56	0.96	0.34
[2, 3, 6, 7, 8]	0.74	0.90	0.33
[1, 5, 6, 7, 8]	0.65	0.93	0.33

[2, 3, 5, 6, 7]	0.55	0.96	0.32
[0, 2, 4, 7, 8]	0.53	0.96	0.31
[1, 4, 5, 7, 8]	0.58	0.94	0.30
[0, 2, 3, 6, 7]	0.76	0.89	0.30
[0, 4, 6, 7, 8]	0.71	0.90	0.30
[0, 2, 5, 6, 7]	0.55	0.94	0.28
[0, 2, 4, 6, 7]	0.58	0.93	0.26
[1, 2, 6, 7, 8]	0.52	0.94	0.26
[0, 2, 6, 7, 8]	0.79	0.87	0.25
[2, 4, 6, 7, 8]	0.58	0.92	0.22
[0, 1, 6, 7, 8]	0.55	0.92	0.19
[0, 3, 5, 6, 7, 8]	0.73	0.96	0.48
[0, 4, 5, 6, 7, 8]	0.56	0.97	0.38
[3, 4, 5, 6, 7, 8]	0.55	0.97	0.37
[2, 3, 5, 6, 7, 8]	055	0.96	0.32
[0, 2, 3, 6, 7, 8]	0.61	0.93	0.29

Table D in S1 file Results pharmacophore screening literature set.

Feature set	Recall	Precision	MCC
[0, 1, 6, 7, 8]	0.69	0.74	0.61
[1, 4, 6, 7, 8]	0.73	0.65	0.57
[0, 1, 4, 7, 8]	0.62	0.74	0.55
[3, 4, 6, 7, 8]	0.65	0.70	0.55
[1, 3, 4, 6, 7]	0.65	0.70	0.55
[4, 5, 6, 7, 8]	0.54	0.81	0.55
[2, 4, 6, 7, 8]	0.54	0.79	0.53
[0, 1, 4, 6, 7]	0.69	0.63	0.53
[0, 3, 4, 7, 8]	0.58	0.72	0.51
[2, 5, 6, 7, 8]	0.54	0.74	0.49
[0, 4, 6, 7, 8]	0.62	0.65	0.48
[0, 3, 6, 7, 8]	0.62	0.65	0.48
[0, 1, 3, 4, 6]	0.54	0.72	0.48
[0, 1, 3, 4, 7]	0.58	0.67	0.46
[0, 3, 4, 6, 7]	0.73	0.52	0.46
[0, 3, 4, 6, 8]	0.58	0.65	0.45
[0, 1, 3, 6, 7]	0.62	0.59	0.43
[0, 1, 4, 6, 7, 8]	0.58	0.87	0.35
[0, 1, 3, 4, 6, 7]	0.54	0.87	0.32
[0, 3, 4, 6, 7, 8]	0.54	0.74	0.15

Table E in S1 file Results pharmacophore screening in which lab and literature sets are combined.

Feature set	Recall	Precision	MCC
[0, 3, 5, 6, 8]	0.63	0.97	0.44
[0, 3, 5, 7, 8]	0.62	0.97	0.44
[0, 4, 5, 6, 8]	0.51	0.97	0.43
[3, 5, 6, 7, 8]	0.66	0.95	0.42
[0, 4, 5, 7, 8]	0.51	0.96	0.41
[1, 4, 5, 7, 8]	0.54	0.94	0.39
[1, 5, 6, 7, 8]	0.59	0.93	0.39
[0, 3, 5, 6, 7]	0.64	0.92	0.38
[0, 4, 5, 6, 7]	0.54	0.93	0.38
[4, 5, 6, 7, 8]	0.66	0.91	0.37
[0, 5, 6, 7, 8]	0.68	0.90	0.37
[2, 3, 6, 7, 8]	0.64	0.90	0.36
[0, 1, 3, 7, 8]	0.55	0.91	0.36
[2, 5, 6, 7, 8]	0.60	0.89	0.35
[1, 3, 6, 7, 8]	0.67	0.88	0.34
[0, 3, 4, 7, 8]	0.61	0.88	0.34
[0, 2, 3, 6, 7]	0.63	0.88	0.34
[0, 2, 6, 7, 8]	0.67	0.87	0.33
[1, 3, 4, 6, 7]	0.61	0.88	0.33
[0, 1, 6, 7, 8]	0.59	0.88	0.33
[3, 4, 6, 7, 8]	0.67	0.86	0.33
[2, 4, 6, 7, 8]	0.57	0.88	0.32
[0, 3, 6, 7, 8]	0.83	0.82	0.32
[0, 3, 4, 6, 8]	0.59	0.86	0.31
[0, 1, 4, 6, 7]	0.66	0.84	0.31
[0, 4, 6, 7, 8]	0.68	0.81	0.29
[0, 1, 3, 6, 7]	0.58	0.82	0.27
[0, 3, 4, 6, 7]	0.67	0.77	0.26
[0, 3, 5, 6, 7, 8]	0.61	0.97	0.44
[0, 2, 3, 6, 7, 8]	0.51	0.94	0.39

22 **Table F in S1 file** Results list of pharmacophore screening with the blocker set.

Blocker	Enantiomer	Feature set
4'-fluoro-6-methoxyflavanone	S	[1, 2, 6, 7, 8]
6,3'-dimethoxyflavanone	R	[1, 2, 6, 7, 8]
6,3'-dimethoxyflavanone	S	[1, 2, 6, 7, 8]
4'-fluoro-6-methoxyflavanone	R	[0, 2, 6, 7, 8]
4'-fluoro-6-methoxyflavanone	S	[0, 2, 6, 7, 8]
6,3'-dimethoxyflavanone	R	[0, 2, 6, 7, 8]
6,3'-dimethoxyflavanone	S	[0, 2, 6, 7, 8]
4'-fluoro-6-methoxyflavanone	R	[0, 1, 6, 7, 8]
4'-fluoro-6-methoxyflavanone	S	[0, 1, 6, 7, 8]
6,3'-dimethoxyflavanone	R	[0, 1, 6, 7, 8]
6,3'-dimethoxyflavanone	S	[0, 1, 6, 7, 8]
6-methoxyflavanone	S	[0, 1, 6, 7, 8]
4'-fluoro-6-methoxyflavanone	S	[0, 1, 2, 7, 8]
6,3'-dimethoxyflavanone	S	[0, 1, 2, 7, 8]
4'-fluoro-6-methoxyflavanone	S	[0, 1, 2, 6, 8]
6,3'-dimethoxyflavanone	S	[0, 1, 2, 6, 8]
4'-fluoro-6-methoxyflavanone	S	[0, 1, 2, 6, 7]
6,3'-dimethoxyflavanone	R	[0, 1, 2, 6, 7]
6,3'-dimethoxyflavanone	S	[0, 1, 2, 6, 7]
4'-fluoro-6-methoxyflavanone	S	[0, 1, 2, 6, 7, 8]
6,3'-dimethoxyflavanone	R	[0, 1, 2, 6, 7, 8]
6,3'-dimethoxyflavanone	S	[0, 1, 2, 6, 7, 8]

23

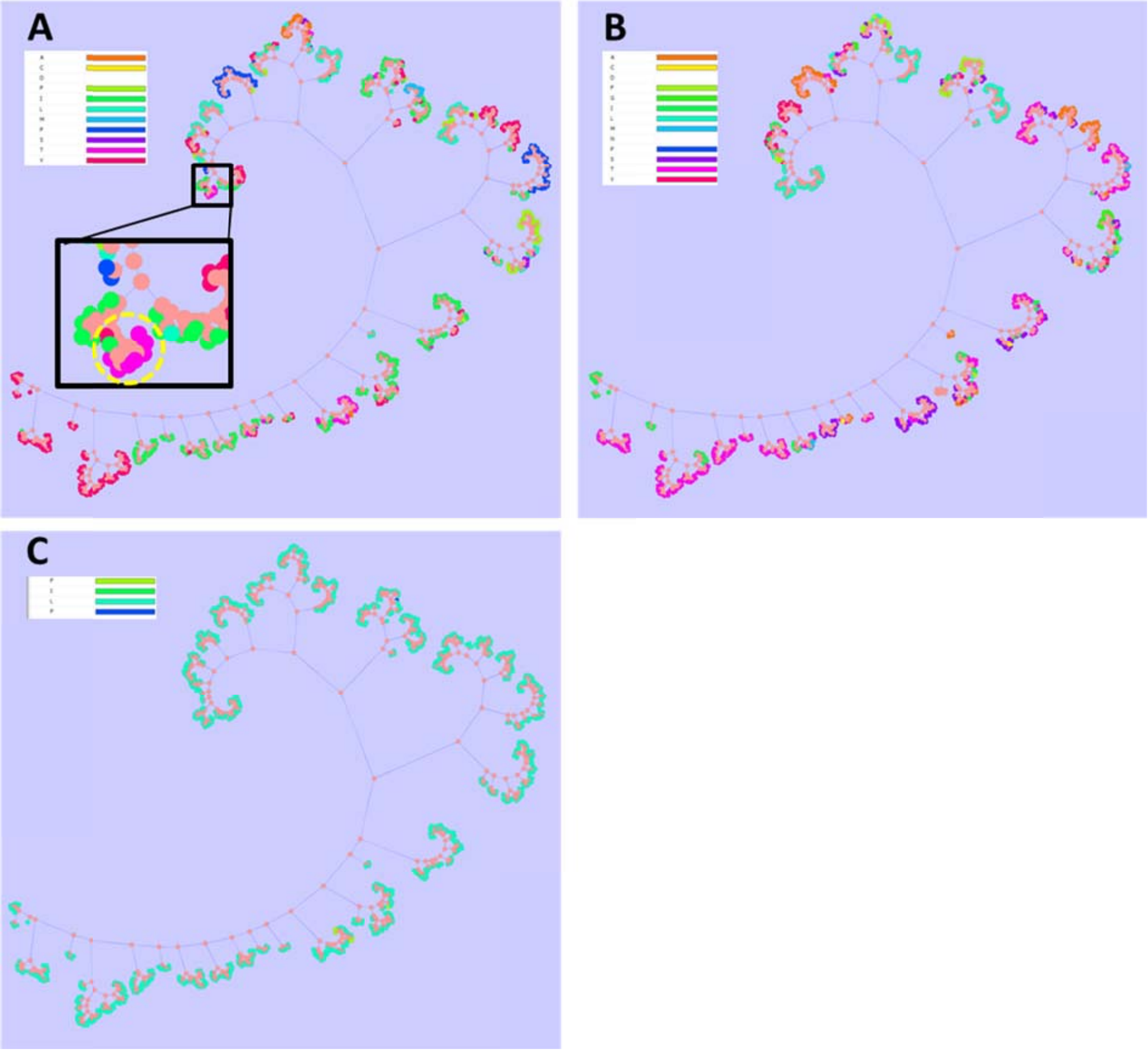

Figure A in S1 file Multiple sequence alignment tree. Each colored node represents a taste receptor. The color of the node is based on the amino acid at a certain position of the alignment. **a)** Zoom-in to receptors selected for the Snooker model building (within the yellow mark). Examples for an **b)** many diverse amino acids at a particular position in the alignment and **c)** identical amino acid at a particular position in the alignment.
